# Antifungalmycin, an antifungal macrolide from *Streptomyces padanus* 702

**DOI:** 10.1007/s13659-011-0037-1

**Published:** 2012-03-03

**Authors:** Yi-Fen Wang, Sai-Jin Wei, Zhi-Ping Zhang, Tong-He Zhan, Guo-Quan Tu

**Affiliations:** 1 State Key Laboratory of Phytochemistry and Plant Resources in West China, Kunming Institute of Botany, Chinese Academy of Sciences, Kunming, 650201 China; 2 Nanchang Key Laboratory of Fermentation Application Technology, China Biological Science and Engineering College of Jiangxi Agriculture University, Nanchang, 330045 China

**Keywords:** antifungalmycin, *Streptomyces padanus* 702, polyene macrolide, biocontrol, antifungal activity, phytopathogen

## Abstract

Two polyene macrolide antibiotics: antifungalmycin (**1**) and fungichromin (**2**) were isolated from the culture mycelia of *Streptomyces padanus* 702 via bioactivity-guided fractionation using various chromatographic procedures. Their structures were elucidated on the basis of spectral analysis, and **1** is a new polyene macrolide. Compounds **1** and **2** showed significant inhibition against *Gibberella zeae* with EC_50_ values of 26.71 and 2.21 µg/mL, *Fusicoccum* sp. (plantain head blight) with EC_50_ values of 23.4 and 3.17 µg/mL, *Mucor* ssp. 8894 with EC_50_ values of 28.80 and 2.11 µg/mL, *Ustilaginoidea virens* with EC_50_ values of 26.72 and 0.21 µg/mL, respectively. This shows that the microbial secondary metabolites **1** and **2** have the potential to be developed as agricultural fungicides for use against *G. zeae, Fusicoccum* sp., *Mucor* ssp. 8894, and *U. virens*.

**Electronic Supplementary Material:**

Supplementary material is available for this article at 10.1007/s13659-011-0037-1 and is accessible for authorized users.

## Introduction

Plant diseases are mainly caused by phytopathogenic fungi, bacteria, and viruses. Among plant diseases, about 70% are caused by phytopathogenic fungi like *Rhizoctonia solani, Aspergillus niger*, *Penicillium notatum* and *Ustilaginoidea virens*[1]. Damping-off caused by *R. solani* is a serious seedling disease that threatens many crops. It causes severe losses in annual plants like vegetables and flowers, in perennial plants such as turf grasses, and in trees grown in nurseries, glasshouses, and gardens. Though these diseases have been successfully controlled by synthetic chemical fungicides, the intensive use of such fungicides causes other problems like fungicide resistance. These fungicides also have environmentally detrimental side effects[1,2]. Microbial metabolites have sophisticated and widely varying structures, which are often associated with highly selective activity against plant pathogens. Antibiotics of microbial origin are likely to show a high rate of degradation in the biosphere and little toxicity to host plants, thus leading to low residue levels. Fungicides of microbial origin such as blasticidin S[3], kasugamycin[4], polyoxin[1], jinggongmycin[1], natamycin[5] and validamycin[6] are being used practically for the control of economically significant plant diseases.

*To whom correspondence should be addressed. E-mail: weisaijin@126.com

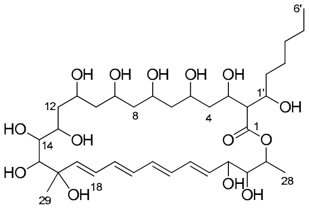

1

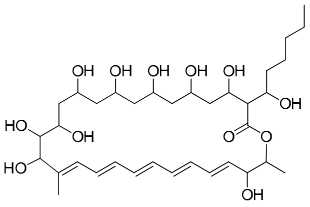

2

Over the last three decades, a variety of antifungal compounds have been produced from various actinomycetes. The genus *Streptomyces* has been the richest source for most antifungal compounds types. Polyene macrolides are widely recognized as one of the representative groups of actinomycete metabolites from the *Streptomyces* species that possess potent antifungal and antiprotozoal activities[7–9]. As a group, polyene macrolides remain widely used in the most commonly available drugs for human fungal infections, including the well-known nystatin and amphotericin B[10]. Targeting *Fusarium* wilt in cotton is a leading reason for *Streptomyces* antibiotic production research for agricultural application. The producing strain *S. padanus* 702 was isolated from a soil strain of *Streptomyces* and was found to produce powerful antifungal activities against various pathogens[11]. However, the precise substances that exhibit these antifungal activities are still

Springer unknown.

Incubation of the strain, followed by extraction and activity-guided separation of the secondary metabolites, yielded the two polyene macrolides. The objectives of this study were to isolate and characterize antifungal substances that may be active against plant pathogenic fungi. The analysis of substances was carried out using various chromatographic procedures and NMR mass spectral analysis.

## Results and Discussion

The screening of antifungal antibiotics from *S. padanus* 702 produced two compounds. Compound **1** was obtained as a yellow powder. Negative FABMS of **1** showed the molecular ion $[M - H]^-$ at *m/z* 703 and high-resolution FABMS revealed the molecular formula $C_{35}H_{59}O_{14}$ (*m/z*: 703.3903, calcd 703.3904), in accord with the $^{13}C$ NMR and DEPT spectra. The IR spectrum suggested the presence of hydroxyl groups (3506 $cm^{-1}$), carbonyl (1721 $cm^{-1}$), and double bonds (1637 $cm^{-1}$). Comparison of the $^1H$ and $^{13}C$ NMR data of **1** (Table 1) and those of fungichromin (**2**)[12] showed that they have the same skeleton. The H-28 signal appears as a unique methyl doublet, and the COSY spectrum gave H-27, H-26, H-25. From H-25, the COSY spectrum gave H-24. Also from the COSY spectrum, H-17 could be assigned because it couples to only one other proton in the polyene region, H-18. $^1H$-$^1H$ COSY, HMQC, and HMBC experiments also determined the assignment of all of the signals of the protons and carbons except for those in the middle part of the 1,3-polyol (C5–C10) and polyene (C19–C23) chains (Figure 1). Comparison of the $^1H$ and $^{13}C$ NMR data of **1** and those of fungichromin showed that main differences were: the absence of two olefinic methine-carbons in **1,** and the presence of two oxygen-bearing carbons (one oxygen-bearing methine and one oxygen-bearing quaternary carbon) in **1**. This suggests the presence of two additional hydroxyl groups and the lack of one double bond in **1**. This is confirmed by its FAB mass spectra. The negative FABMS of **1** gave a quasi-molecular ion peak at *m/z* 703, suggesting an increase of 34 mass units, compared to that of fungichromin. The H-29 signal as a unique methyl single was correlated with C-15 ($\delta_C$ 84.6, CH), C-16 ($\delta_C$ 83.3, C), and C-17 ($\delta_C$ 139.6, CH), which indicated that one hydroxyl is located at C-16. The correlations observed between H-26 ($\delta_H$ 4.35, m), H-25 ($\delta_H$ 5.13, br. s), and H-27 ($\delta_H$ 5.60, t, $J$ = 7.1 Hz) in COSY spectra suggested that the additional hydroxyl group was located at C-25. This was confirmed by the correlations between H-27, C-25, C-26, and C-28 in HMBC experiments. The combination of COSY, HMQC, and HMBC experiments determined the gross structure of **1**, including the ester linkage between C-1 and C-27. Consequently, the structure of **1** was elucidated, and named antifungalmycin. Fungichromin (**2**) is the prototype of a group of 28-membered macrolides and is characterized by pentaene in the molecule. Antifungalmycin (**1**) is a new derivative of fungichromin, has the same size of the macrocyclic lactone ring, and shows an absence of one carbon-to-carbon double bond.

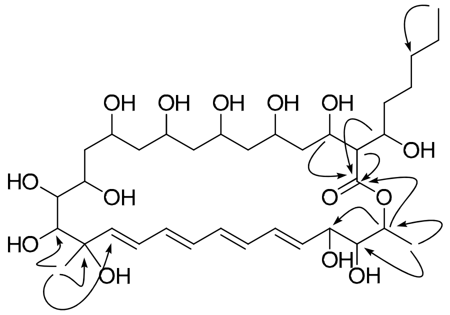

**Figure 1.** The key HMBC correlations of compound **1**

Antibiotics have received a great deal of interest for potential application in agriculture, biomedicine, biotechnology, and the food industry. Those produced by actinomycetes (such as genus *Streptomyces*), which have been used in agriculture to control plant pathogenic diseases over the past century, have received particular attention. Among these, polyene macrolides is an important class of antifungal antibiotics, which are of specific interest due to their little fungicide resistance.

Polyene macrolide antibiotics are characterized by the number of conjugated carbon-to-carbon double bonds in the molecule, the size of the macrocyclic lactone ring, and the presence or absence of a hexosamine sugar or aromatic moiety in the molecule[9]. Amphotericin B (AmB), nystatin, and filipin III are classified into heptaene, tetraene, and pentaene antibiotics on the basis of the number of continued-conjugated double bonds.

**Table 1. The $^1H$ and $^{13}C$ NMR spectral data for 1 in pyridine-$d_5$ (500 MHz and 125 MHz, $J$ in Hz, $\delta$ in ppm)**

| No. | $\delta_H$ | $\delta_C$ | No. | $\delta_H$ | $\delta_C$ |
|---|---|---|---|---|---|
| 1 | | 172.9, C | 19 | 6.33 (dd, 14.5, 11.0) | 135.2, CH |
| 2 | 3.24 (t, 8.0) | 59.6, CH | 20 | 6.46 (dd, 14.6, 11.0) | 131.8, CH |
| 3 | 5.00 (m) | 72.2, CH | 21 | 6.40 (m) | 134.1, CH |
| 4 | 2.25 (m) | 42.1, $CH_2$ | 22 | 6.72 (dd, 14.8, 10.8) | 131.5, CH |
| 5 | 4.58 (m) | 72.7, CH | 23 | 7.03 (dd, 14.8, 10.8) | 131.8, CH |
| 6 | 1.90 (m) | 45.3, $CH_2$ | 24 | 6.68 (m) | 134.5, CH |
| 7 | 4.67 (m) | 71.8, CH | 25 | 5.13 (br. s) | 72.8, CH |
| 8 | 1.87 (m) | 45.6, $CH_2$ | 26 | 4.35 (m) | 77.3, CH |
| 9 | 4.50 (m) | 71.6, CH | 27 | 5.60 (t, 7.1) | 72.4, CH |
| 10 | 1.84 (m) | 43.4, $CH_2$ | 28 | 1.75 (d, 6.2) | 18.4, $CH_3$ |
| 11 | 4.80 (m) | 69.8, CH | 29 | 1.65 (s) | 24.0, $CH_3$ |
| 12 | 2.43 (m) | 42.5, $CH_2$ | 1′ | 4.56 (m) | 72.4, CH |
| 13 | 4.13 (m) | 76.3, CH | 2′ | 2.01 (m) | 35.9, $CH_2$ |
| 14 | 4.35 (m) | 84.3, CH | 3′ | 1.88 (m) | 25.8, $CH_2$ |
| 15 | 4.61 (d, 5.8) | 84.6, CH | 4′ | 1.27 (m) | 32.2, $CH_2$ |
| 16 | | 83.3, C | 5′ | 1.25 (m) | 23.0, $CH_2$ |
| 17 | 6.02 (d, 15.0) | 139.6, CH | 6′ | 0.79 (t, 6.8) | 14.3, $CH_3$ |
| 18 | 6.75 (m) | 127.7, CH | | | |

The antifungal activity of two polyene macrolides from the culture broth of *S. padanus* 702 was evaluated using mould, yeast and 8 species of plant pathogenic fungi via the mycelial growth rate method. For the bioassay using mould and yeast, the final concentrations of antifungalmycin (**1**) were 0, 6.25, 12.5, 25, 50, and 100 $\mu$g/mL, and the final concentrations of fungichromin (**2**) were 0, 0.8125, 1.625, 3.25, 6.5, and 13 $\mu$g/mL. The minimum inhibitory concentrations (MIC, $\mu$g/mL) of **1** and **2** against *A. niger* and *Candida utilis* were 25 and 1.625 $\mu$g/mL, respectively.

As shown by the mycelial growth rate method, these two polyene macrolides showed potential antifungal activities, including but not limited to the following: *R. solani, Helminthosporium sigmoideum*, *Magnaporthe grisea, P. notatum, Fusicoccum* sp. (plantain head blight), *G. zeae*, and *Mucor* ssp. 8894 (Table 2). Their indoor toxicity (the toxicity tested in the laboratory) was tested on mycelia growth of *U. virens* using the mycelium dry weight method. Compounds **1** and **2** had some inhibitory effect on mycelia growth of *U. virens;* their respective $EC_{50}$ values were 26.72 $\mu$g/mL and 0.21 $\mu$g/mL (Table 2).

The antifungal activities of two pure polyene macrolides against 8 plant pathogenic fungi were evaluated using the hyphal growth inhibition assay. In comparison to antifungalmycin (**1**), fungichromin (**2**) showed a higher level of inhibitory activities against all phytopathogenic fungi. Because antifungalmycin is similar to fungichromin, compound **1** has the potential to be developed as agricultural fungicides.

Antifungalmycin (**1**) and fungichromin (**2**) showed potential inhibition against *P. notatum,* with $EC_{50}$ values of 18.24 and 1.33 $\mu$g/mL. This is in accord with previous reports that polyenes exhibited toxicity against *P. notatu*[13–16]. Fungichromin exhibited a stronger toxicity against *P. notatum* than antifungalmycin did. This indicates that the pentaene was the central element in the molecule, and both polyenes may be active against *P. notatum*.

Many microbial secondary metabolites were used for the control of plant diseases caused by *R. solani*[17,18], *H. sigmoideum*[19,20], and *M. grisea*[21–24]. However, there are no reports that polyenes exhibited toxicity against *R. solani, H. sigmoideum,* and *Magnaporthe grisea*. We found that two polyenes: antifungalmycin (**1**) and fungichromin (**2**), showed a strong toxicity against *R. solani,* with $EC_{50}$ values of 6.20 and 1.47 $\mu$g/mL. The polyenes also showed potential inhibition of *H. sigmoideum,* with $EC_{50}$ values of 11.05 and 2.53 $\mu$g/mL. Polyenes **1** and **2** demonstrated potential inhibition of *Magnaporthe grisea*, with $EC_{50}$ values of 15.24 and 3.25 $\mu$g/mL. Our findings suggest that like many microbial secondary metabolites, polyene macrolides also have the potential to be core compounds to develop agricultural fungicides against *R. solani, H. sigmoideum,* and *Magnaporthe grisea*.

According to our knowledge, there are no reports that microbial secondary metabolites have been used for the control of plant diseases caused by *Fusicoccum* sp. (plantain head blight), *G. zeae, Mucor* ssp. 8894, or *U. virens*. Our former study found that the extract of *S. padanus* 702 exhibited toxicity against *G. zeae*[11]. In this study, two polyene macrolides (**1** and **2**) isolated from extract of *S. padanus* 702, showed potential inhibition against *G. zeae*, with $EC_{50}$ values of 26.71 and 2.21 $\mu$g/mL. Further, with $EC_{50}$ values of 23.4 and 3.17 $\mu$g/mL, antifungalmycin (**1**) and fungichromin (**2**) showed potential inhibition of *Fusicoccum* sp. (plantain head blight). They also showed potential inhibition of *Mucor* ssp. 8894, with $EC_{50}$ values of 28.80 and 2.11 $\mu$g/mL. For *U. virens*, macrolides **1** and **2** also showed inhibitory activities, with $EC_{50}$ values of 26.72 and 0.21 $\mu$g/mL. This study found that the microbial secondary metabolites, antifungalmycin (**1**) and fungichromin (**2**), have the potential to be developed as agricultural fungicides, and specifically as products for the treatment of phytopathogenic fungi.

## Experimental Section

**General Experimental Procedures**. The melting point was measured with a Fisher-Johns micromelting point apparatus.

**Table 2. Effects of compounds 1 and 2 against 8 species of plant pathogenic fungi**

| plant pathogenic fungi | compd. | toxicity regression equation | correlation coefficient | $EC_{50}$ $\mu$g/ml | $EC_{90}$ $\mu$g/ml |
|---|---|---|---|---|---|
| *R. solani* | **1** | Y=4.8524+0.3848X | r=0.9844 | 1.47 | 41.02 |
| | **2** | Y=2.525+1.3585X | r=0.9990 | 6.20 | 15.94 |
| | natamycin | Y=4.9312+1.315X | r=0.9947 | 1.13 | 10.64 |
| *H. sigmoideum* | **1** | Y=1.5136+1.4513X | r=0.9988 | 11.05 | 26.72 |
| | **2** | Y=4.4019+0.6443X | r=0.9816 | 2.53 | 18.49 |
| | natamycin | Y=3.9937+1.9872X | r=0.9907 | 3.21 | 14.17 |
| *Magnaporthe grisea* | **1** | Y=1.9331+1.1035X | r=0.9702 | 15.24 | 37.72 |
| | **2** | Y=3.5677+1.1555X | r=0.9768 | 3.45 | 10.47 |
| | natamycin | Y=3.9355+1.7275X | r=0.9966 | 4.13 | 22.81 |
| *P. notatum* | **1** | Y=2.1222+0.8534X | r=0.9911 | 18.24 | 66.48 |
| | **2** | Y=4.663+1.1928X | r=0.9310 | 1.33 | 3.88 |
| | natamycin | Y=5.6827+1.9979X | r=0.9920 | 0.46 | 1.99 |
| *Fusicoccum* sp. (plantain head blight) | **1** | Y=1.8314+0.978X | r=0.9914 | 23.40 | 94.66 |
| | **2** | Y=3.7797+1.0575X | r=0.9849 | 3.17 | 10.65 |
| | natamycin | Y=4.8576+2.1410X | r=0.9961 | 1.17 | 4.62 |
| *G. zeae* | **1** | Y=1.6067+1.033X | r=0.9866 | 26.71 | 92.34 |
| | **2** | Y=4.4572+0.6823X | r=0.9358 | 2.21 | 14.50 |
| | natamycin | Y=5.0577+1.1331X | r=0.9914 | 0.89 | 12.02 |
| *Mucor* ssp. 8894 | **1** | Y=1.3604+1.083X | r=0.9894 | 28.80 | 94.07 |
| | **2** | Y=3.9484+1.412X | r=0.9201 | 2.11 | 5.22 |
| | natamycin | Y=3.9355+1.7275X | r=0.9966 | 4.13 | 22.81 |
| *U. virens* | **1** | Y=2.3157+0.817X | r=0.9240 | 26.72 | 128.27 |
| | **2** | Y=5.3136+0.1979X | r=0.8906 | 0.21 | 133.1 |
| | natamycin | Y=4.5431+2.4658X | r=0.9967 | 1.53 | 5.07 |

$^1H$, $^{13}C$, and 2D NMR spectra were recorded on a Bruker AM 400 NMR, and a DRX-500 spectrometer with TMS as the internal standard. FAB-MS data were obtained with a VG AutoSpec 3000 spectrometer. UV spectra were obtained using a Shimadzu double-beam 210A spectrophotometer. The IR (KBr) spectra were recorded on a Bio-Rad FTS-135 spectrometer. Chemical shifts were reported as parts per million ($\delta$), using the residual $C_5D_5N$ as an internal standard and coupling constants ($J$) in hertz. $^1H$ and $^{13}C$ NMR assignments were supported by $^1H$-$^1H$ COSY, HMQC, and HMBC experiments. The negative FABMS and HRFABMS spectra were recorded on a VG AutoSpec 3000 spectrometer in *m/z* (rel. %). Optical rotation was measured on a Horiba SEAP-300 sensitive polarimeter. Silica gel (200-300 mesh) for column chromatography, and $GF_{254}$ for TLC were obtained from the Qindao Marine Chemical Factory, Qindao, China. HPLC separations were performed on a Shimadzu LC-20AT system, detected by a UV detector at 203 nm, and equipped with a YMC semipreparative C18 column (10 $\mu$m, 10 × 250 mm) running with a flow rate of 3 mL/min. All chemicals used in the study, such as methanol (MeOH), chloroform ($CHCl_3$), acetonitrile ($CH_3CN$), and acetone, were of analytical grade.

**Microorganism and Media.** The Addressing the threat of *Fusarium* wilt in cotton is the target goal of agriculturally-applicable research on the antibiotic production of *Streptomyces*. The producing strain 702 was isolated from a soil strain of *Streptomyces* and deposited at Nanchang Key Laboratory of Fermentation Application and Technology, Biological Science and Engineering College of Jiangxi Agriculture University, China. Based on its partial sequence of the 16S rRNA gene (GenBank accession number JF437715) and the other character, strain 702 was identified to be *Streptomyces padanus* by Prof. Guo-Quan Tu. *A. niger*, *C. utilis*, *R. solani*, *H. sigmoideum*, *Magnaporthe grisea*, *P. notatum, Fosicoccum sp., G. zeae, Mucor* ssp. 8894 and *U. virens* were stored at the Nanchang Key Laboratory of Fermentation Application and Technology, Biological Science and Engineering College of Jiangxi Agricultural University, China. All organism cultures were grown on potato dextrose agar (PDA) slants. The seed liquid culture medium (g/L) for *S. padanus* 702 was comprised of 20 g of sucrose, 30 g of corn powder, 10 g of soybean powder, 4 g of yeast extract, 6 g of $CaCO_3$, and 4 mL of soya bean oil. The fermentation medium (g/L) was comprised of 2.5 g of cornstarch, 30 g of corn powder, 20 g of dextrose, 1 g of peptone, 15 g of soybean powder, 5 g of $CaCl_2$, 10 mL of soya bean oil, 0.3 g of $KH_2PO_4$, and 5 g of $KNO_3$; all were stirred with distilled water to 1.0 L. The pH was adjusted to 8.0 and the medium was sterilized at 121 °C for 30 min.

**Culture Material and Fermentation.** A spore of the pure *S. padanus* 702 strain in 20% glycerol was grown on PDA for 5–7 days. The spore suspension ($T_{660nm}$ = 80%) was prepared from a culture grown in seed liquid culture medium in 400 mL flasks held at 30 °C for 48 h on a rotary shaker (200 rpm) and used as seed stock. The medium was inoculated with 2.5% (v/v) spore inoculum containing $2 \times 10^9$ c.f.u./ mL. Seed cultivation was carried out in 2000-mL Erlenmayer Flasks with 400 ml of the seed liquid culture medium held at 30 °C, while on a rotary shaker at 200 rpm for 48 h. For large production of culture filtrates, *S. padanus* 702 was grown in a 50 L fermentor (model: GUCS50) containing 3 L seed liquid cultures. It was aerated at 30 L/min, and stirred at 200 rpm at 30 °C for 36 h. These cultures were grown in a 500 L fermentor (model: GUJS500C), aerated at 300 L/min, and stirred at 200 rpm at 30 °C for 6 days. The 6-day-old fermentation was then extracted, concentrated, and stored at 4 °C in the dark for antibiosis studies and chemical analysis.

**Extraction and Isolation.** From a large-scale fermentation (200 L, antifungal activity unit: 10000 mg/L), mycelium was extracted with alcohol for 24 h from the culture filtrate by filtration at room temperature. After concentration *in vacuo*, the aqueous concentrate was partitioned in AcOEt (3 × 60 L) for 3 h each time. The combined extracts were concentrated under reduced pressure to yield 68 g of a brown gum. The AcOEt fraction was fixed on silica gel (680 g) and fractionated through flash chromatography, eluting with a gradient mixture of chloroform-methanol (from 100:0 to 0:100), and giving nine fractions (A–I). Fraction D was subjected to column chromatography (CC) on silica gel, using chloroform-methanol (90:10) as elution to afford five sub-fractions[25]. The sub-fraction showed strong antifungal activity and was chosen for further purification. This fraction was successively subjected to open column chromatography using silica gel (gradient chloroform-methanol) and Sephadex LH-20 (MeOH). Final purification of the macrocyclic polyene derivations was achieved by preparative RP-HPLC: Aglient Zorbax ODS 250 × 21 mm, flow rate 10 mL/min, gradient MeOH-$H_2O$ 60% in 13 min, 60–70% in 20 min and 70-100% MeOH in 8 min, and UV detection at 210 nm. This afforded two compounds, **1** (250 mg) and **2** (560 mg).

**Antifungalmycin (1):** $C_{35}H_{60}O_{14}$, yellow powder; mp. 170–173.5℃; $[\alpha]_D^{23}$ = + 4.34 ($c$ 0.1, MeOH), UV $\lambda_{max}$ (MeOH): 318, 303, 290, 211 nm; IR (KBr) $\nu_{max}$: 3506, 2926, 2855, 1721, 1637, 1384, 1241, 1099 $cm^{-1}$; Negative FAB-MS *m/z* (%): 703 $[M - H]^-$ (100). Negative HRFABMS *m/z*: 703.3903 ($[M - H]^-$, calcd. 703.3904). $^1H$ and $^{13}C$ NMR spectral data see Table 1.

**Minimum Inhibitory Concentration.** The minimum inhibitory concentrations of the purified compounds were determined using the following procedure. PDA powder was dissolved in distilled water to a final concentration of 50 g/L and then sterilized at 121 °C for 15 min. The sterilized PDA solution was placed in a water bath, and the temperature was cooled and maintained at 55 to 60 °C. The antifungal agent stock solution (1 mL, dissolved in water) was mixed with the PDA solution (9 mL) to produce a series of different final concentrations. Drug-free agar, containing only water, was used as a control. The mixtures of antifungal agent and PDA solutions were poured directly into Petri dishes (10 cm diameter). After the plates were cooled to room temperature, 0.01 ml of freshly-made fungal suspension ($2 \times 10^8$ c.f.u./mL or the 5–8 mm agar block) was inoculated onto the agar of each Petri dish (4 test organisms in each Petri dish). The plates were incubated at 25°C. The inhibitory effects of compounds **1** and **2** on the growth of test organisms were evaluated after 2–3 days of incubation. The lowest concentration of antibiotics that completely inhibited the growth of the microorganisms was considered to be a minimum inhibitory concentration (MIC). Natamycin was used as positive control against plant pathogenic fungi.

**Bioassay of Antibiotic.** Purified compounds were tested for antibiosis against plant pathogenic fungi using the mycelial growth rate method. The antifungal agent stock solution (1 ml, dissolved in sterile water) was mixed with the PDA solution (9 mL) to produce a series of different final concentrations (Table 2). Drug-free agar containing only sterile water was used as a control. Each Petri dish was then inoculated with an agar block (5–8 mm diameter) containing the mycelial mats of plant pathogenic fungi in the center of the dish. The inhibition percentage was obtained after treatment for 3–4 days at 25 °C, until the growth in the control plates reached the plate edges. The $EC_{50}$ value was obtained from the regression equations of their toxicities, which were calculated using the least square method. Antifungal activity was recorded as follows:

Growth Inhibition Rate (%) = $[1 - (Da - D)/(Db - D)]\times 100\%$

Where *Da* is the growth diameter in the treatment, *Db* is the growth diameter in the control and *D* is the agar block (5–8 mm diameter).

Each treatment consisted of three replicates. The experiment was repeated twice.

## Electronic Supplementary Material

Supplementary material is available in the online version of this article at http://dx.doi.org/ 10.1007/s13659-011-0037-1 and is accessible for authorized users.

## Acknowledgments

This work was supported by the National Natural Science Foundation (20872148 and 30960011), and the Natural Science Foundation of Jiangxi Province (2009GZN0030). The authors are grateful to the analytical group of the Laboratory of Phytochemistry, Kunming Institute of Botany, Chinese Academy of Sciences for measuring NMR, MS, and IR data.

**Open Access** This article is distributed under the terms of the Creative Commons Attribution License which permits any use, distribution, and reproduction in any medium, provided the original author(s) and source are credited.

## Electronic supplementary material

Supplementary material, approximately 442 KB.
