# Supplementary material for: Antifungalmycin, an antifungal macrolide from Streptomyces padanus 702
Source: Nat Prod Bioprospect. 2012 Mar 3;2(1):41–5. doi: 10.1007/s13659-011-0037-1 (PMC4131570; doi:10.1007/s13659-011-0037-1)
Supplement: Supplementary file 1 — Supplementary material, approximately 442 KB. [file 13659_2011_37_MOESM1_ESM.pdf]

Electronic Supplementary Material

Antifungalmycin, an antifungal macrolide from *Streptomyces padanus* 702

Yi-Fen WANG,[a,†] Sai-Jin WEI,[b,†] Zhi-Ping ZHANG,[b] Tong-He ZHAN,[b] and Guo-Quan TU[b,]*

[a]State Key Laboratory of Phytochemistry and Plant Resources in West China, Kunming Institute of Botany, Chinese Academy of Sciences, Kunming 650201, China

[b]China Biological Science and Engineering College of Jiangxi Agriculture University, Nanchang Key Laboratory of Fermentation Application Technology, Nanchang 330045, China

[†]These authors contributed equally to this work.

Received 23 November 2011; Accepted 20 February 2012

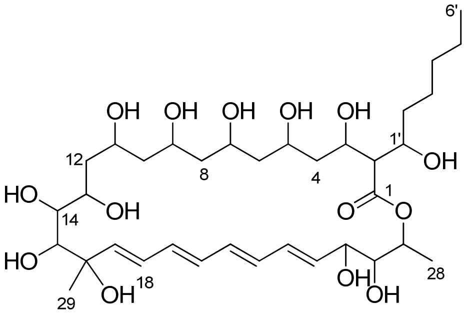

1

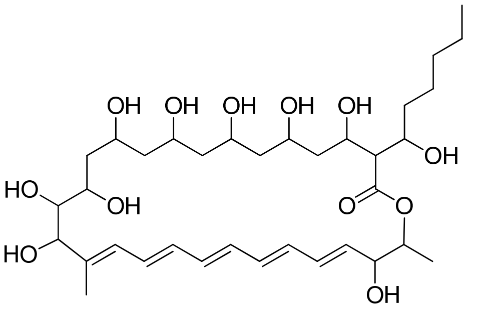

2

Structures of compounds **1** and **2**.

*To whom correspondence should be addressed. E-mail: weisaijin@126.com

dzp12 H

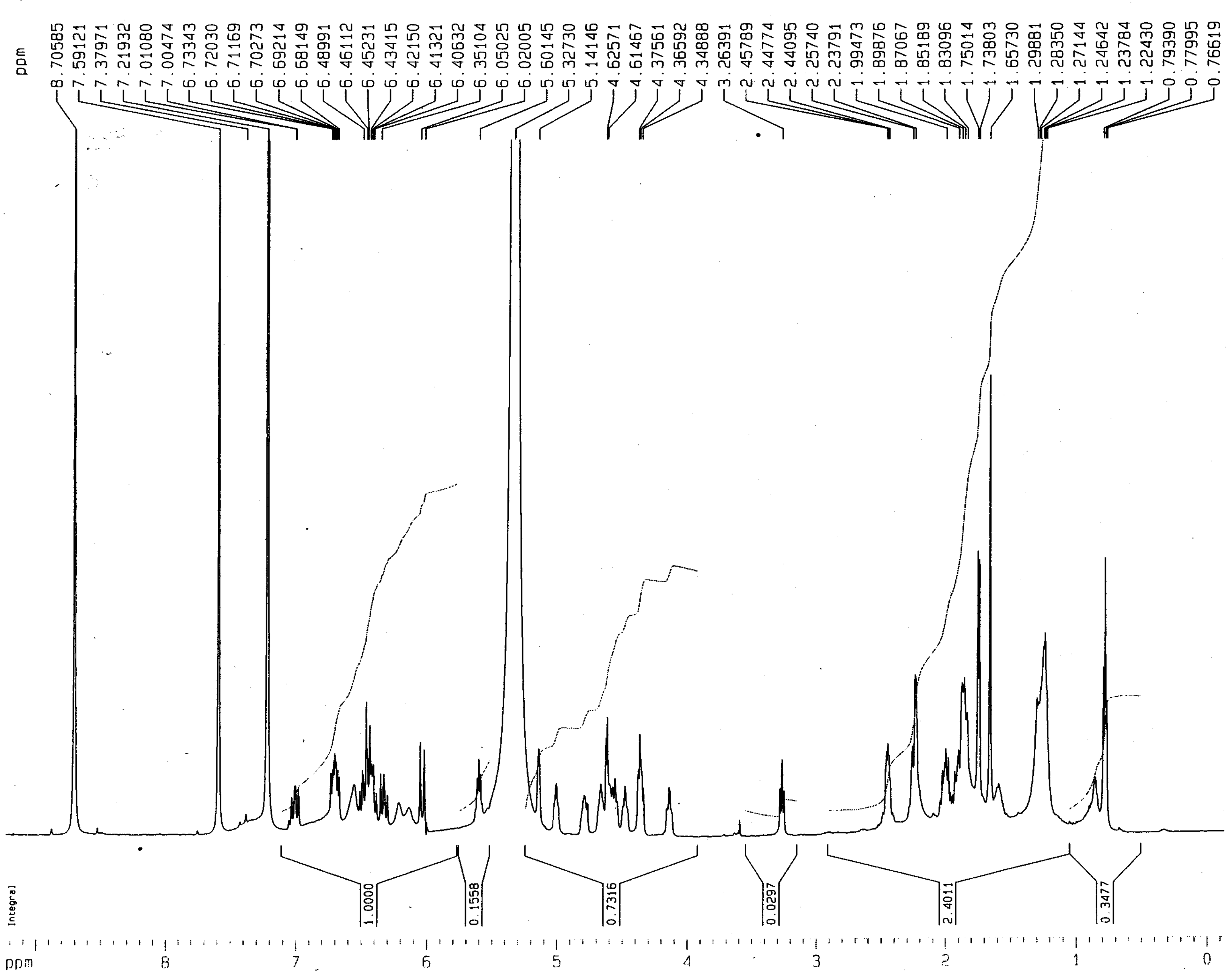

Current Data Parameters

NAME	dzp12
EXPNO	21
PROCNO	1

F2 - Acquisition Parameters

Date_	20091022
Time	13.29
INSTRUM	spect
PROBHD	5 mm BBI 1H-BB
PULPROG	zg
TD	32768
SOLVENT	Acetone
NS	1
DS	0
SWH	7507.507 Hz
FIDRES	0.229111 Hz
AQ	2.1823988 sec
RG	25.4
DW	66.600 usec
DE	6.00 usec
TE	0.0 K
D1	1.00000000 sec
MCREST	0.00000000 sec
MCWRK	0.01500000 sec

======== CHANNEL f1 ========

NUC1	1H
P1	9.20 usec
PL1	-1.00 dB
SFO1	500.0330002 MHz

F2 - Processing parameters

SI	16384
SF	500.0299976 MHz
WDW	EM
SSB	0
LB	0.30 Hz
GB	0
PC	1.00

1D NMR plot parameters

CX	22.00 cm
CY	230.00 cm
F1P	9.236 ppm
F1	4618.03 Hz
F2P	-0.129 ppm
F2	-64.41 Hz
PPMCM	0.42565 ppm/cm
HZCM	212.83804 Hz/cm

dzp12 c13

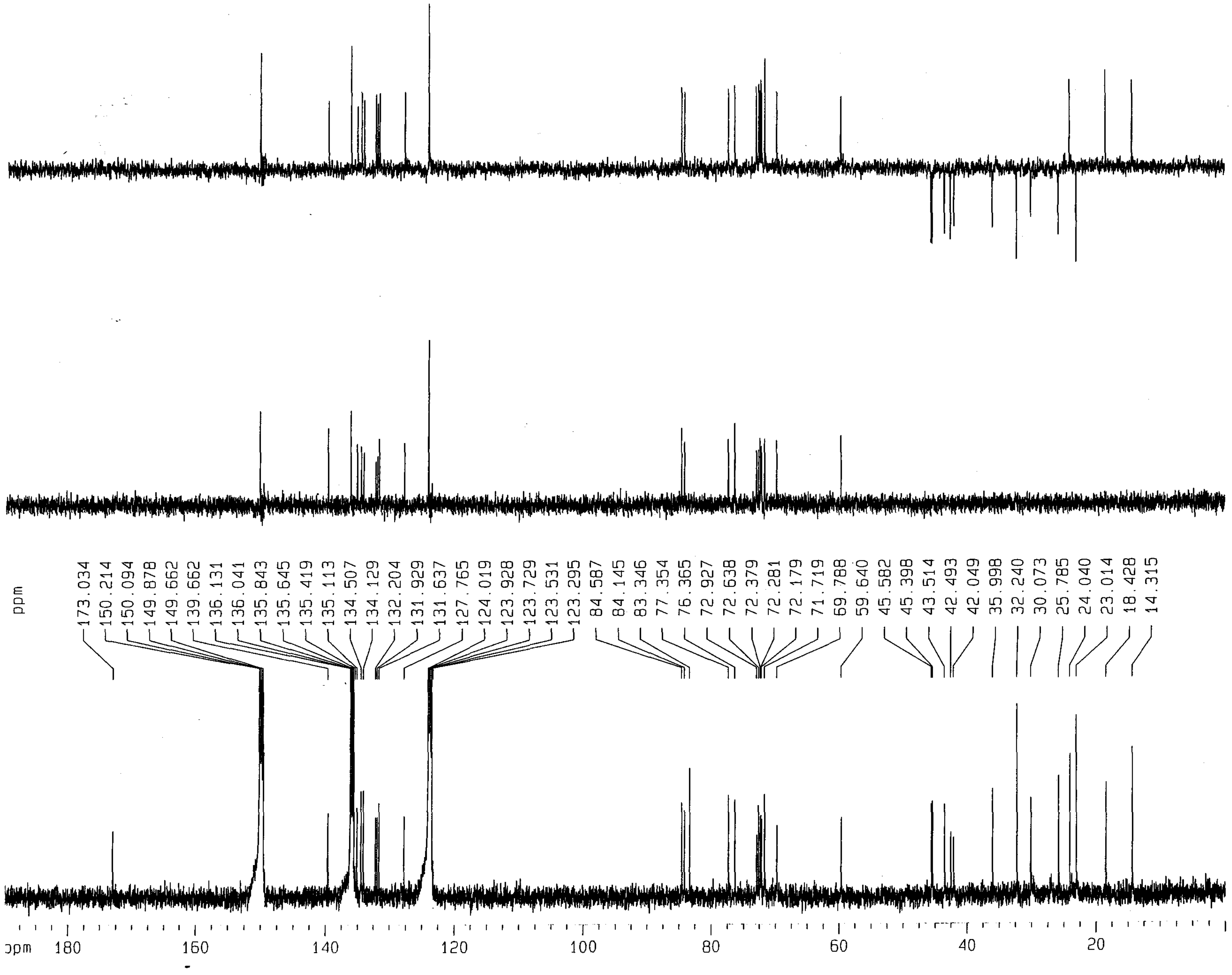

Current Data Parameters

NAME	dzp12
EXPNO	22
PROCNO	1

F2 - Acquisition Parameters

Date_	20091029
Time	19.35
INSTRUM	spect
PROBHD	5 mm DUL 13C-1
PULPROG	zgdc
TD	32768
SOLVENT	Pyr
NS	480
DS	0
SWH	28985.508 Hz
FIDRES	0.884567 Hz
AQ	0.5652980 sec
RG	2298.8
DW	17.250 usec
DE	6.00 usec
TE	0.0 K
D1	3.00000000 sec
d11	0.03000000 sec
MCREST	0.00000000 sec
MCWRK	0.01500000 sec

======== CHANNEL f1 ========

NUC1	13C
P1	5.90 usec
PL1	0.00 dB
SFO1	125.7467261 MHz

======== CHANNEL f2 ========

CPDPRG2	waltz16
NUC2	1H
PCPD2	84.00 usec
PL2	-4.00 dB
PL12	18.00 dB
SFO2	500.0325001 MHz

F2 - Processing parameters

SI	16384
SF	125.7326006 MHz
WDW	EM
SSB	0
LB	1.00 Hz
GB	0
PC	1.30

1D NMR plot parameters

CX	22.00 cm
CY	65.00 cm
F1P	190.000 ppm
F1	23889.19 Hz
F2P	-0.000 ppm
F2	-0.00 Hz
PPMCM	8.63636 ppm/cm
HZCM	1085.87244 Hz/cm

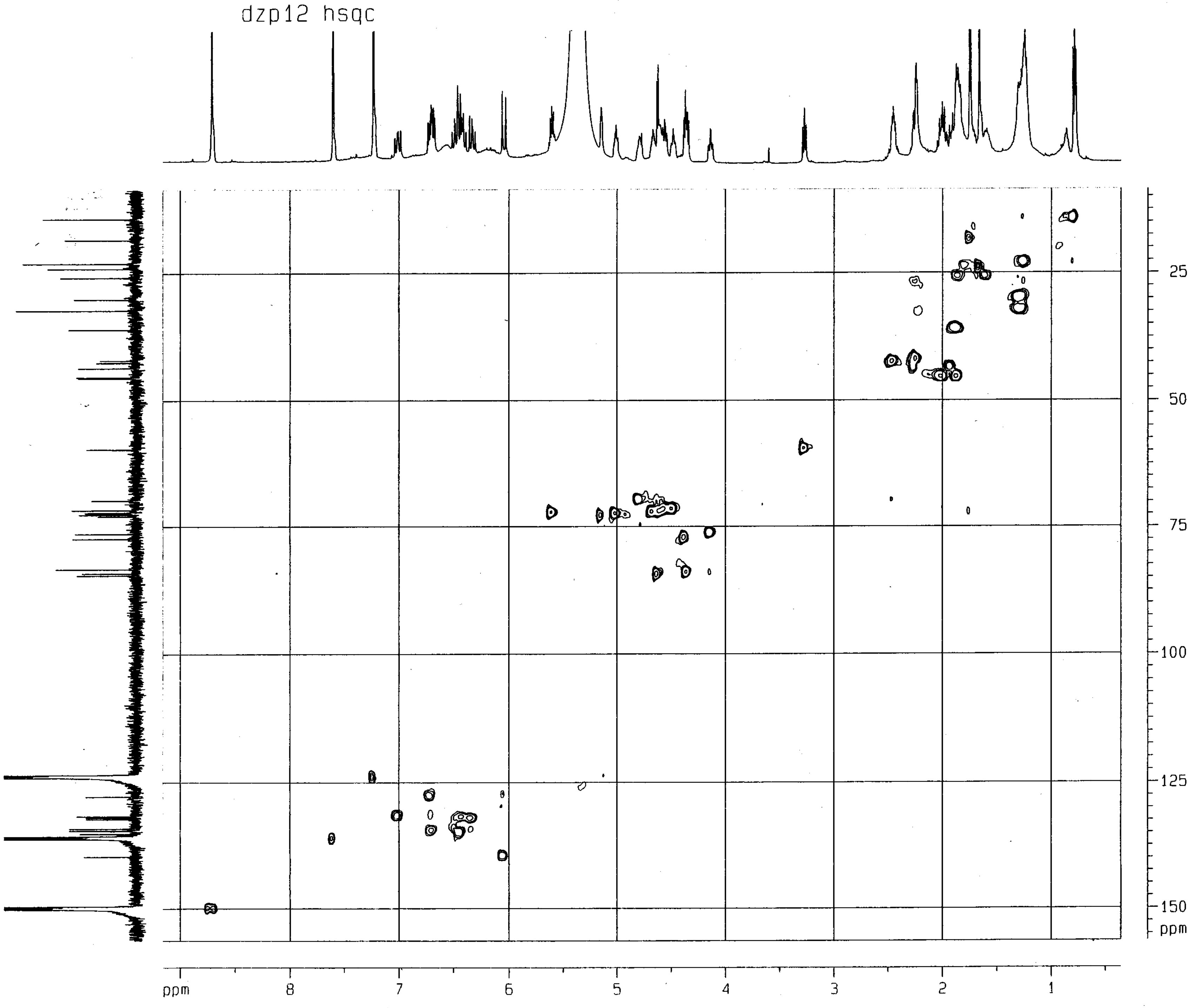

Current Data Parameters

NAME	dzp12
EXPNO	33
PROCNO	1

F2 - Acquisition Parameters

Date_	20091113
Time	9.21
INSTRUM	spect
PROBHD	5 mm BBI 1H-BB
PULPROG	hsqcetgpsi2
TD	1024
SOLVENT	Pyr
NS	2
DS	16
SWH	6009.615 Hz
FIDRES	5.868765 Hz
AQ	0.0852468 sec
RG	9195.2
DW	83.200 usec
DE	6.00 usec
TE	0.0 K
CNST2	145.0000000
d0	0.00000300 sec
D1	1.00000000 sec
d4	0.00172414 sec
d11	0.03000000 sec
d13	0.00000400 sec
D16	0.00020000 sec
D24	0.00086207 sec
DELTA	0.00127440 sec
DELTA1	0.00120800 sec
DELTA2	0.00006207 sec
DELTA3	0.00052414 sec
IN0	0.00002425 sec
MCREST	0.00000000 sec
MCWRK	0.16666700 sec
ST1CNT	128

======== CHANNEL f1 ========

NUC1	1H
P1	9.20 usec
p2	18.40 usec
P28	2000.00 usec
PL1	-1.00 dB
SFO1	500.0325502 MHz

======== CHANNEL f2 ========

CPDPRG2	garp
NUC2	13C
P3	12.00 usec
p4	24.00 usec
PCPD2	75.00 usec
PL2	-1.00 dB
PL12	15.50 dB
SFO2	125.7428283 MHz

====== GRADIENT CHANNEL =====

GPNAM1	SINE.100
GPNAM2	SINE.100
GPNAM3	SINE.100
GPNAM4	SINE.100
GPX1	0.00 %
GPX2	0.00 %
GPX3	0.00 %
GPX4	0.00 %
GPY1	0.00 %
GPY2	0.00 %
GPY3	0.00 %
GPY4	0.00 %
GPZ1	80.00 %
GPZ2	20.10 %
GPZ3	11.00 %
GPZ4	-5.00 %
P16	1000.00 usec
P19	600.00 usec

F1 - Acquisition parameters

ND0	2
TD	128
SFO1	125.7416 MHz
FIDRES	161.082474 Hz
SW	163.976 ppm
FnMODE	Echo-Antiecho

F2 - Processing parameters

SI	1024
SF	500.0299870 MHz
WDW	QSINE
SSB	2
LB	0.00 Hz
GB	0
PC	1.40

F1 - Processing parameters

SI	1024
MC2	echo-antiecho
SF	125.7313692 MHz
WDW	QSINE
SSB	2
LB	0.00 Hz
GB	0

2D NMR plot parameters

CX2	18.00 cm
CX1	14.00 cm
F2PLO	9.163 ppm
F2LO	4582.02 Hz
F2PHI	0.361 ppm
F2HI	180.44 Hz
F1PLO	156.408 ppm
F1LO	19665.38 Hz
F1PHI	8.594 ppm
F1HI	1080.49 Hz
F2PPMCM	0.48903 ppm/cm
F2HZCM	244.53189 Hz/cm
F1PPMCM	10.55816 ppm/cm
F1HZCM	1327.49219 Hz/cm

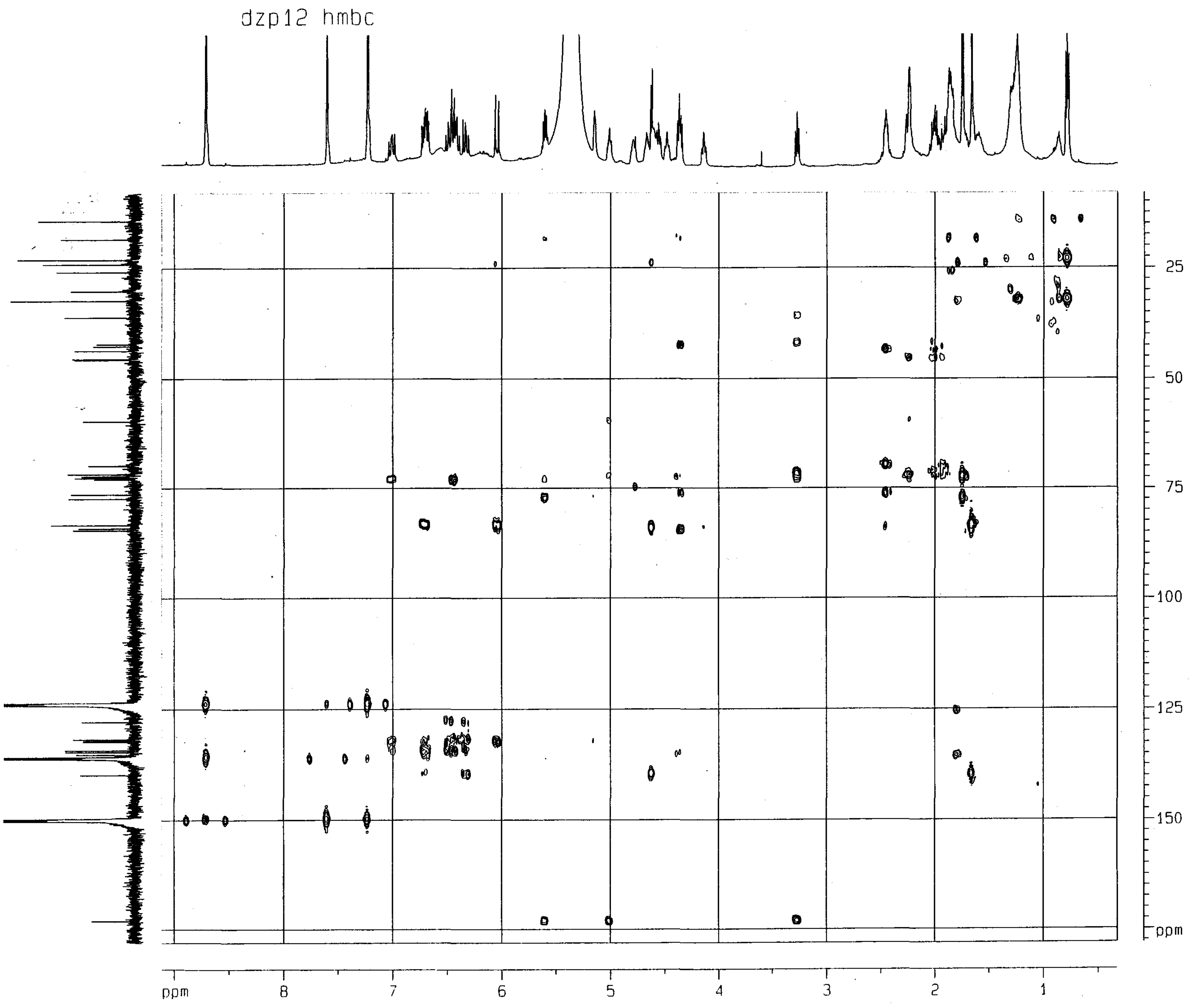

Current Data Parameters

NAME	dzp12
EXPNO	32
PROCNO	. 1

F2 - Acquisition Parameters

Date_	20091113
Time	7.41
INSTRUM	spect
PROBHD	5 mm BBI 1H-BB
PULPROG	inv4gplplrndqf
TD	2048
SOLVENT	Pyr
NS	32
DS	16
SWH	6009.615 Hz
FIDRES	2.934382 Hz
AQ	0.1704436 sec
RG	18390.4
DW	83.200 usec
DE	6.00 usec
TE	0.0 K
CNST2	145.0000000
d0	0.00000300 sec
D1	1.20000005 sec
d2	0.00344828 sec
D6	0.08340000 sec
d13	0.00000400 sec
D16	0.00020000 sec
IN0	0.00002115 sec
MCREST	0.00000000 sec
MCWRK	1.20000005 sec

======== CHANNEL f1 ========

NUC1	1H
P1	9.20 usec
p2	18.40 usec
PL1	-1.00 dB
SFO1	500.0325502 MHz

======== CHANNEL f2 ========

NUC2	13C
P3	12.00 usec
PL2	-1.00 dB
SFO2	125.7447143 MHz

====== GRADIENT CHANNEL =====

GPNAM1	SINE.100
GPNAM2	SINE.100
GPNAM3	SINE.100
GPX1	0.00 %
GPX2	0.00 %
GPX3	0.00 %
GPY1	0.00 %
GPY2	0.00 %
GPY3	0.00 %
GPZ1	50.00 %
GPZ2	30.00 %
GPZ3	40.10 %
P16	1000.00 usec

F1 - Acquisition parameters

ND0	2
TD	128
SFO1	125.7447 MHz
FIDRES	184.692673 Hz
SW	188.005 ppm
FnMODE	QF

F2 - Processing parameters

SI	1024
SF	500 0299942 MHz
WDW	SINE
SSB	0
LB	0.00 Hz
GB	0
PC	1.40

F1 - Processing parameters

SI	512
MC2	QF
SF	125.7326164 MHz
WDW	SINE
SSB	0
LB	0.00 Hz
GB	0

2D NMR plot parameters

CX2	18.00 cm
CX1	14.00 cm
F2PLO	9.114 ppm
F2LO	4557.19 Hz
F2PHI	0 323 ppm
F2HI	161.48 Hz
F1PLO	178.112 ppm
F1LO	22394 53 Hz
F1PHI	8 083 ppm
F1HI	1016.32 Hz
F2PPMCM	0.48838 ppm/cm
F2HZCM	244.20584 Hz/cm
F1PPMCM	12 14494 ppm/cm
F1HZCM	1527.01477 Hz/cm

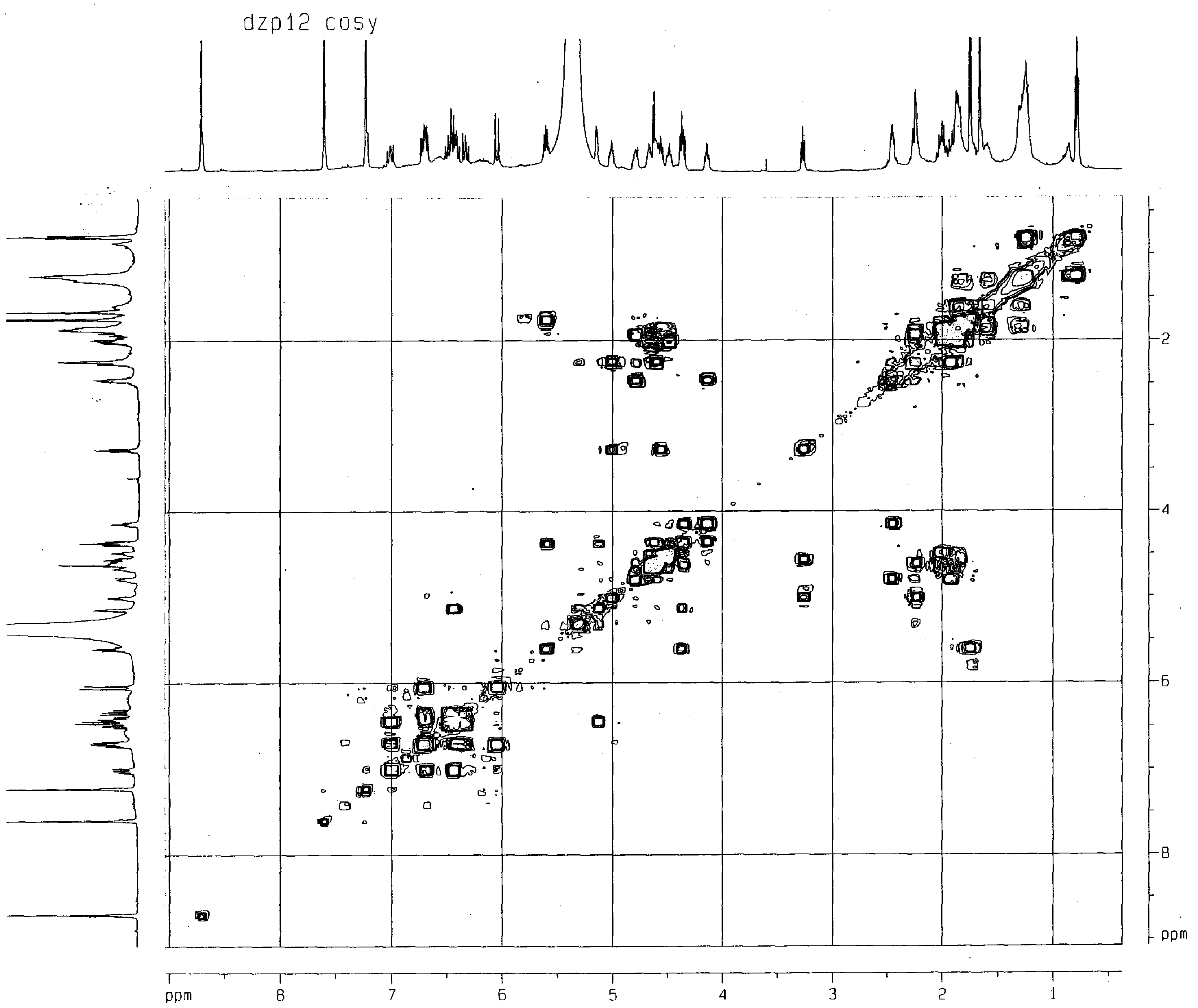

Current Data Parameters

NAME	dzp12
EXPNO	34
PROCNO	1

F2 - Acquisition Parameters

Date_	20091113
Time	9.26
INSTRUM	spect
PROBHD	5 mm BBI 1H-BB
PULPROG	cosygpmfqf
TD	1024
SOLVENT	Pyr
NS	1
DS	16
SWH	6009.615 Hz
FIDRES	5.868765 Hz
AQ	0.0852468 sec
RG	23170.5
DW	83.200 usec
DE	6.00 usec
TE	0.0 K
D0	0.00000300 sec
D1	1.00000000 sec
d13	0.00000400 sec
D16	0.00020000 sec
IN0	0.00016665 sec
MCREST	0.00000000 sec
MCWRK	1.00000000 sec

======== CHANNEL f1 ========

NUC1	1H
P1	9.20 usec
PL1	-1.00 dB
SFO1	500.0325502 MHz

====== GRADIENT CHANNEL =====

GPNAM1	sine.100
GPNAM2	sine.100
GPNAM3	sine.100
GPX1	0.00 %
GPX2	0.00 %
GPX3	0.00 %
GPY1	0.00 %
GPY2	0.00 %
GPY3	0.00 %
GPZ1	16.00 %
GPZ2	12.00 %
GPZ3	40.00 %
P16	1000.00 usec

F1 - Acquisition parameters

ND0	1
TD	128
SFO1	500.0318 MHz
FIDRES	46.879688 Hz
SW	12.000 ppm
FnMODE	QF

F2 - Processing parameters

SI	1024
SF	500.0299957 MHz
WDW	SINE
SSB	0
LB	0.00 Hz
GB	0
PC	1.00

F1 - Processing parameters

SI	1024
MC2	QF
SF	500.0291911 MHz
WDW	SINE
SSB	0
LB	0.00 Hz
GB	0

2D NMR plot parameters

CX2	18.00 cm
CX1	14.00 cm
F2PLO	9.040 ppm
F2LO	4520.48 Hz
F2PHI	0.379 ppm
F2HI	189.33 Hz
F1PLO	9.079 ppm
F1LO	4539.69 Hz
F1PHI	0.336 ppm
F1HI	168.16 Hz
F2PPMCM	0.48121 ppm/cm
F2HZCM	240.61937 Hz/cm
F1PPMCM	0.62447 ppm/cm
F1HZCM	312.25223 Hz/cm

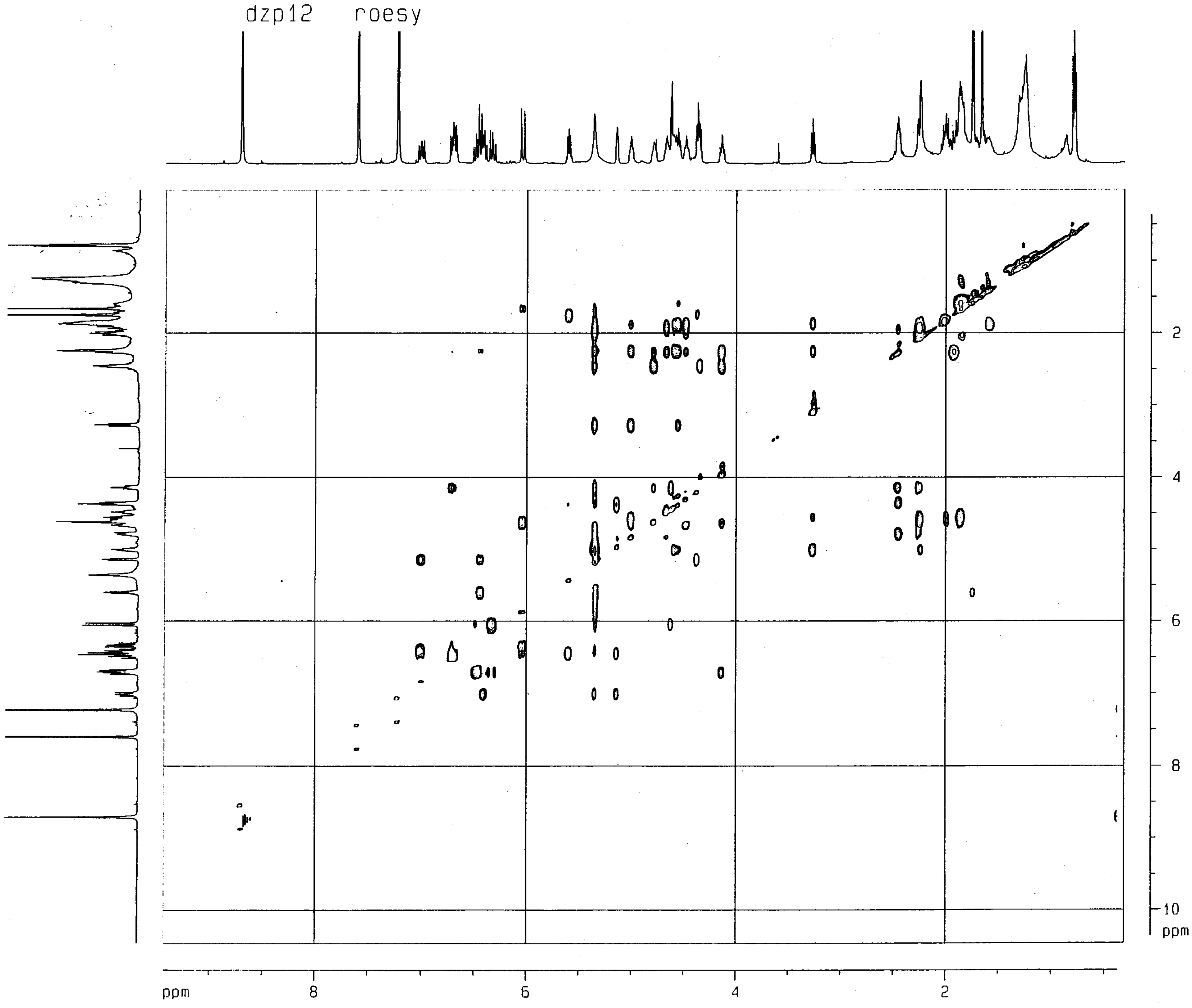

Current Data Parameters

NAME	dzp12
EXPNO	28
PROCNO	1

F2 - Acquisition Parameters

Date_	20091110
Time	18.04
INSTRUM	spect
PROBHD	5 mm BBI 1H-BB
PULPROG	croesyphpr
TD	1024
SOLVENT	DMSO
NS	8
DS	4
SWH	5000.000 Hz
FIDRES	4.882812 Hz
AQ	0.1024500 sec
RG	96
DW	100.000 usec
DE	6.00 usec
TE	0.0 K
d0	0.00008828 sec
D1	1.20000005 sec
d11	0.03000000 sec
d12	0.00002000 sec
d13	0.00000400 sec
IN0	0.00019999 sec
MCREST	0.00000000 sec
MCWRK	0.00999999 sec
ST1CNT	90

======== CHANNEL f1 ========

NUC1	1H
P1	9.20 usec
P15	320000.00 usec
PL1	-1.00 dB
PL9	70.00 dB
PL11	22.00 dB
SFO1	500.0326802 MHz

F1 - Acquisition parameters

ND0	1
TD	147
SFO1	500.0321 MHz
FIDRES	34.015732 Hz
SW	10.000 ppm
FnMODE	States-TPPI

F2 - Processing parameters

SI	1024
SF	500.0299953 MHz
WDW	QSINE
SSB	2
LB	0.00 Hz
GB	0
PC	1.00

F1 - Processing parameters

SI	1024
MC2	States-TPPI
SF	500.0294155 MHz
WDW	QSINE
SSB	2
LB	0.00 Hz
GB	0

2D NMR plot parameters

CX2	18.00 cm
CX1	14.00 cm
F2PLO	9.431 ppm
F2LO	4715.80 Hz
F2PHI	0.316 ppm
F2HI	157.92 Hz
F1PLO	10.464 ppm
F1LO	5232.48 Hz
F1PHI	0.023 ppm
F1HI	11.25 Hz
F2PPMCM	0.50640 ppm/cm
F2HZCM	253.21539 Hz/cm
F1PPMCM	0.74585 ppm/cm
F1HZCM	372.94458 Hz/cm

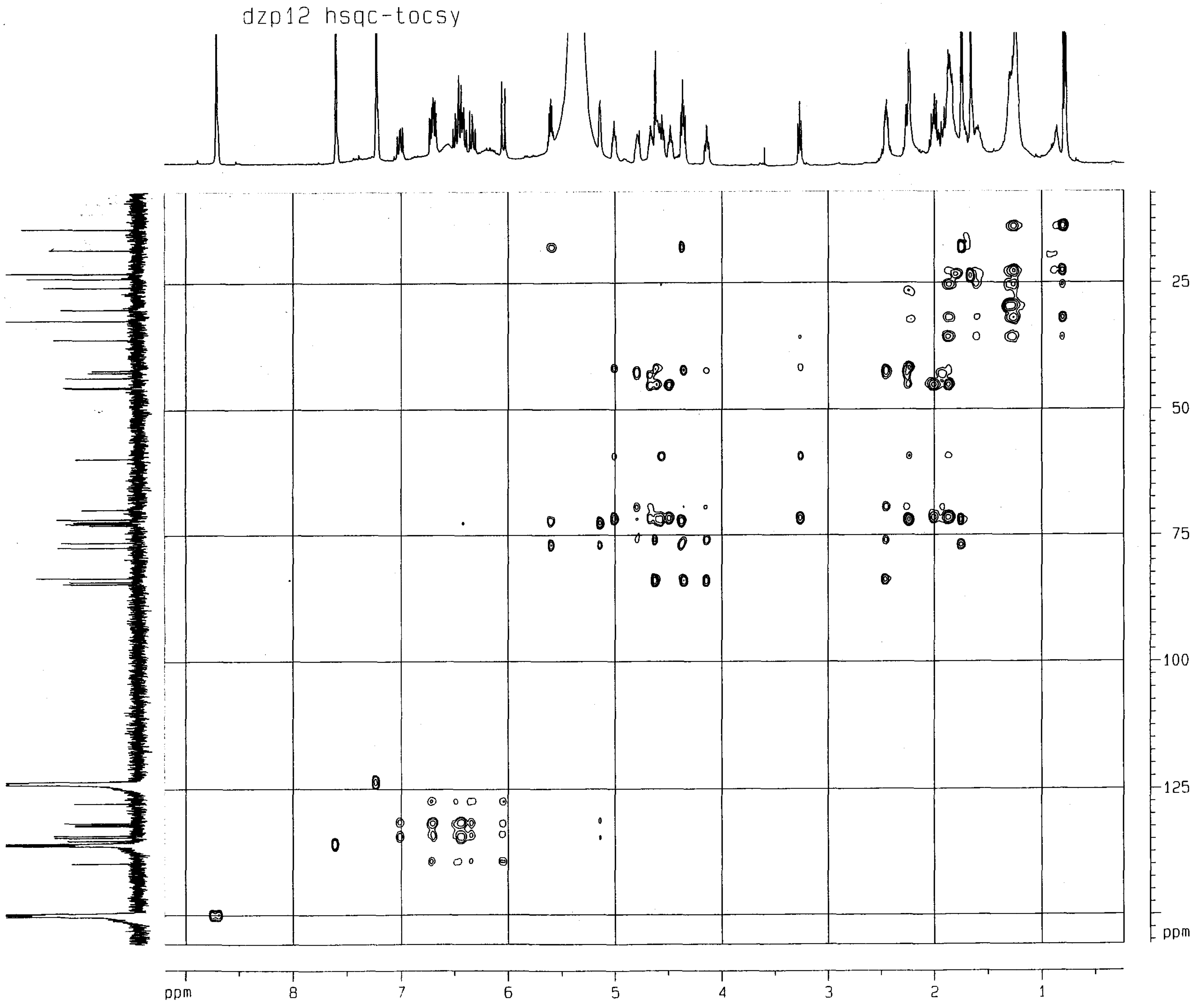

Current Data Parameters

NAME	dzp12	
EXPNO	36	
PROCNO	1	

F2 - Acquisition Parameters

Date_	20091113	
Time	10.28	
INSTRUM	spect	
PROBHD	5 mm BBI 1H-BB	
PULPROG	invietgpml	
TD	1024	
SOLVENT	Pyr	
NS	18	
DS	16	
SWH	6510.417	Hz
FIDRES	6.357829	Hz
AQ	0.0786932	sec
RG	9195.2	
DW	76.800	usec
DE	6.00	usec
TE	0.0	K
CNST2	145.0000000	
d0	0.00000300	sec
D1	1.20000005	sec
d4	0.00172414	sec
D9	0.06000000	sec
d11	0.03000000	sec
d12	0.00002000	sec
d13	0.00000400	sec
D16	0.00020000	sec
DELTA	0.00122440	sec
DELTA1	0.00120800	sec
FACTOR1	4	
IN0	0.00002485	sec
l1	24	
MCREST	0.00000000	sec
MCWRK	0.24000001	sec
SCALEF	6	
ST1CNT	112	

======== CHANNEL f1 ========

NUC1	1H	
P1	9.20	usec
p2	18.40	usec
p5	23.34	usec
P6	35.00	usec
p7	70.00	usec
P17	2500.00	usec
P28	2000.00	usec
PL1	-1.00	dB
PL10	11.50	dB
SFO1	500.0326002	MHz

======== CHANNEL f2 ========

CPDPRG2	garp	
NUC2	13C	
P3	12.00	usec
p4	24.00	usec
PCPD2	75.00	usec
PL2	-1.00	dB
PL12	15.50	dB
SFO2	125.7428283	MHz

====== GRADIENT CHANNEL =====

GPNAM1	SINE.100	
GPNAM2	SINE.100	
GPX1	0.00	%
GPX2	0.00	%
GPY1	0.00	%
GPY2	0.00	%
GPZ1	80.00	%
GPZ2	20.10	%
P16	1000.00	usec

F1 - Acquisition parameters

ND0	2	
TD	224	
SFO1	125.7428	MHz
FIDRES	89.824661	Hz
SW	160.015	ppm
FnMODE	Echo-Antiecho	

F2 - Processing parameters

SI	1024	
SF	500.0299901	MHz
WDW	QSINE	
SSB	2	
LB	0.00	Hz
GB	0	
PC	1.40	

F1 - Processing parameters

SI	1024	
MC2	echo-antiecho	
SF	125.7326442	MHz
WDW	QSINE	
SSB	2	
LB	0.00	Hz
GB	0	

2D NMR plot parameters

CX2	18.00	cm
CX1	14.00	cm
F2PLO	9.200	ppm
F2LO	4600.09	Hz
F2PHI	0.236	ppm
F2HI	117.82	Hz
F1PLO	155.855	ppm
F1LO	19596.10	Hz
F1PHI	7.079	ppm
F1HI	890.11	Hz
F2PPMCM	0.49800	ppm/cm
F2HZCM	249.01497	Hz/cm
F1PPMCM	10.62685	ppm/cm
F1HZCM	1336.14185	Hz/cm

Optical rotation measurement

Model : P-1020 (A060460638)

No.	Sample	Mode	Data	Monitor<br>Blank	Temp.<br>Cell<br>Temp Point	Date<br>Comment<br>Sample Name	Light<br>Filter<br>Operator	Cycle Time<br>Integ Time
	DZP8							
No.1	6 (1/3)	Sp.Rot	4.7710	0.0026<br>0.0000	23.7<br>50.00<br>Cell	Tue Oct 20 13:36:42 2009<br>0.00109g/mlMeOH<br>DZP8	Na<br>589nm	2 sec<br>10 sec
No.2	6 (2/3)	Sp.Rot	4.0370	0.0022<br>0.0000	23.7<br>50.00<br>Cell	Tue Oct 20 13:36:55 2009<br>0.00109g/mlMeOH<br>DZP8	Na<br>589nm	2 sec<br>10 sec
No.3	6 (3/3)	Sp.Rot	4.2200	0.0023<br>0.0000	23.6<br>50.00<br>Cell	Tue Oct 20 13:37:08 2009<br>0.00109g/mlMeOH<br>DZP8	Na<br>589nm	2 sec<br>10 sec

+4.3427°

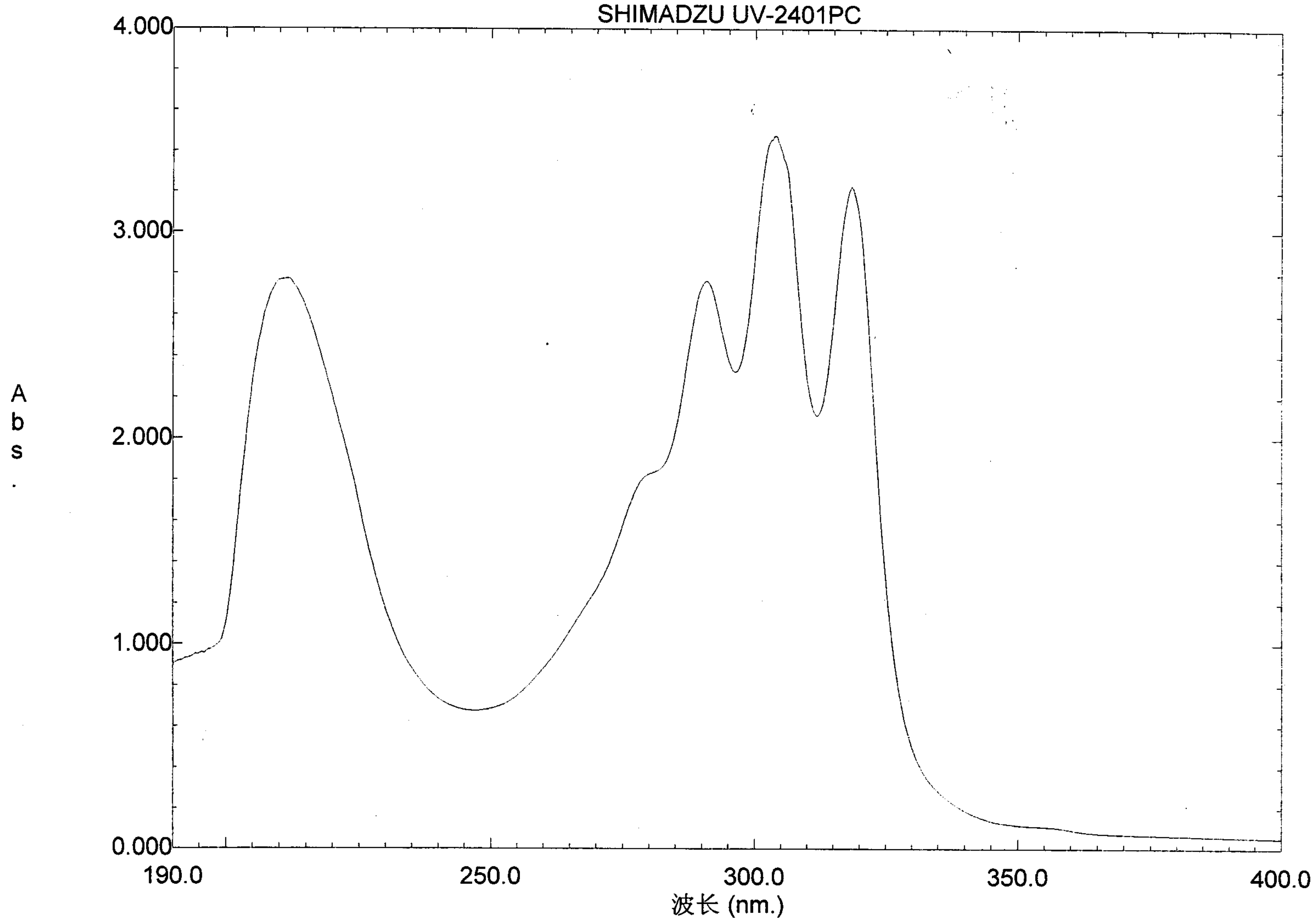

文件名: DZP8

DZP8

创建于: 14:24 09-10-19
数据: 原始

样品浓度：0.0780毫克/毫升
溶剂：甲醇

测量模式: Abs.
扫描速度: 中速
狭缝: 2.0
采样间隔: 0.2

否.	波长 (nm.)	Abs.
1	318.40	3.2265
2	303.60	3.4753
3	290.80	2.7634
4	211.60	2.7731

56: [illegible] 309)

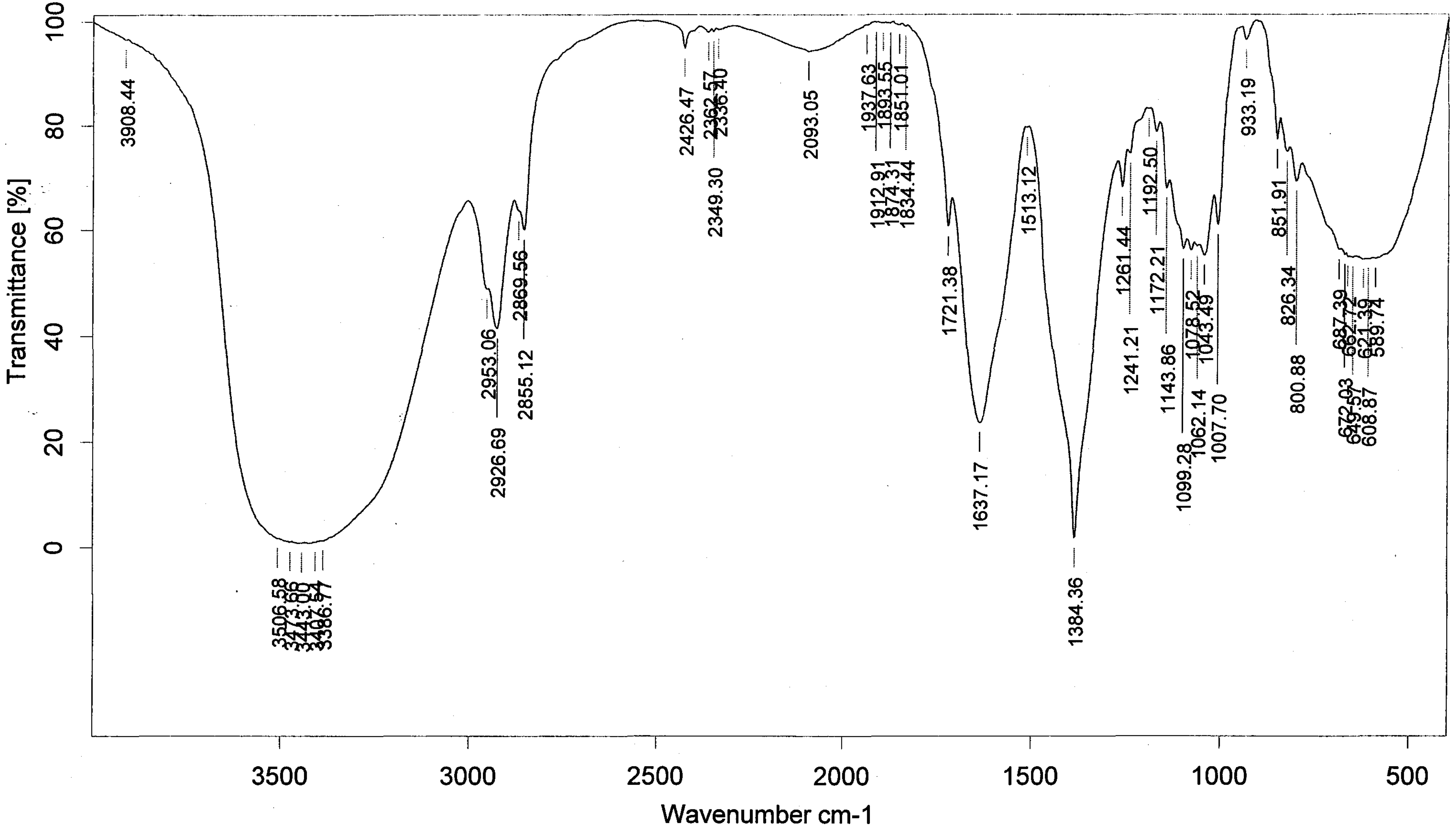

Sample : DZP8		Frequency Range : 399.271 - 3996.57	Measured on : 21/10/2009
Technique : KBr压片	Resolution : 4	Instrument : Tensor27	Sample Scans : 16
Customer : 091021IR	Zerofilling : 2	Acquisition : Double Sided,For	
